# Comparative Respiratory Tract Microbiome Between Carbapenem-Resistant *Acinetobacter baumannii* Colonization and Ventilator Associated Pneumonia

**DOI:** 10.3389/fmicb.2022.782210

**Published:** 2022-03-04

**Authors:** Tingting Xiao, Qian Guo, Yanzi Zhou, Ping Shen, Yuan Wang, Qiang Fang, Mo Li, Shuntian Zhang, Lihua Guo, Xiao Yu, Yulin Liao, Chunhui Wang, Xiaohui Chi, Xiaoyang Kong, Kai Zhou, Beiwen Zheng, Qixia Luo, Yunbo Chen, Huaiqiu Zhu, Yonghong Xiao

**Affiliations:** ^1^State Key Laboratory for Diagnosis and Treatment of Infectious Diseases, National Clinical Research Center for Infectious Diseases, National Medical Center for Infectious Diseases, Collaborative Innovation Center for Diagnosis and Treatment of Infectious Diseases, The First Affiliated Hospital, Zhejiang University School of Medicine, Hangzhou, China; ^2^State Key Laboratory for Turbulence and Complex Systems, Department of Biomedical Engineering, College of Future Technology and Center for Quantitative Biology, Peking University, Beijing, China; ^3^Department of Intensive Care Unit, The First Affiliated Hospital, Zhejiang University School of Medicine, Hangzhou, China; ^4^Shenzhen Institute of Respiratory Diseases, The First Affiliated Hospital (Shenzhen People’s Hospital), Southern University of Science and Technology, Shenzhen, China

**Keywords:** carbapenem-resistant *Acinetobacter baumannii* (CRAB), ventilator-associated pneumonia (VAP), multi-genomics analysis, microbiome, virulence gene

## Abstract

**Background:**

Carbapenem-resistant *Acinetobacter baumannii* (CRAB) is a common cause of ventilator-associated pneumonia (VAP) in intensive care unit (ICU) patients, but its infection and colonization state are difficult to distinguish. If the judgment is wrong, it may aggravate the abuse of antibiotics and further accelerate the evolution of drug resistance. We sought to provide new clues for the diagnosis, pathogenesis and treatment of CRAB VAP based on lower respiratory tract (LRT) microbiota.

**Methods:**

A prospective study was conducted on patients with mechanical ventilation from July 2018 to December 2019 in a tertiary hospital. Multi-genomics studies (16S rRNA amplicon, metagenomics, and whole-genome sequencing [WGS]) of endotracheal deep aspirate (ETA) were performed.

**Results:**

Fifty-two ICU patients were enrolled, including 24 with CRAB VAP (CRAB-I), 22 with CRAB colonization (CRAB-C), and six CRAB-negative patients (infection-free) (CRAB-N). Diversity of pulmonary microbiota was significantly lower in CRAB-I than in CRAB-C or CRAB-N (mean Shannon index, 1.79 vs. 2.73 vs. 4.81, *P* < 0.05). Abundances of 11 key genera differed between the groups. *Acinetobacter* was most abundant in CRAB-I (76.19%), moderately abundant in CRAB-C (59.14%), and least abundant in CRAB-N (11.25%), but its interactions with other genera increased in turn. Metagenomics and WGS analysis showed that virulence genes were more abundant in CRAB-I than in CRAB-C. Multi-locus sequence typing (MLST) of 46 CRAB isolates revealed that the main types were ST208 (30.43%) and ST938 (15.22%), with no difference between CRAB-I and CRAB-C.

**Conclusion:**

Lower respiratory tract microbiota dysbiosis including elevated relative abundance of *Acinetobacter* and reduced bacterial interactions, and virulence enrichment may lead to CRAB VAP.

## Introduction

*Acinetobacter baumannii* (AB) is a ubiquitous microorganism that can contaminate the surface of hospital equipment and colonize skin, wounds, and other parts of patients (Shamsizadeh et al., 2017). Ventilator associated pneumonia (VAP) is a frequent complication in ICUs and is associated with prolonged mechanical ventilation, longer intensive care unit (ICU) stay, and poorer outcomes (Papazian et al., 2020). *Acinetobacter baumannii* is frequently isolated from the respiratory tract in patients with tracheal intubation, which is considered to be a high-risk factor for VAP (Consales et al., 2011; Huang et al., 2018). Yin et al. (2021) found that the prevalence of AB was high (31.7%) among VAP pathogens in 15 teaching hospitals in China from 2007 to 2016. Due to the heavy use of broad-spectrum antibacterial agents in ICU patients, most AB strains were multi-drug resistant or even pan-drug resistant. A survey of hospital-acquired pneumonia (HAP) and VAP conducted from 2007 to 2013 revealed that multidrug-resistant AB (MDRAB) increased yearly and ranked first in some ICU bacterial lists (Hu et al., 2016).

The carbapenem-resistant AB (CRAB) genome encodes various drug-resistance genes and virulence factors (VFs), including efflux pumps, iron acquisition systems, secretion systems, phospholipases, and capsular polysaccharides, which help the bacterium survive antibiotic treatment and colonize in the environment (Harding et al., 2018; Uppalapati et al., 2020). Without effective therapy, the mortality of patients with nosocomial CRAB infection remains high (Xie et al., 2018; Yin et al., 2021). Accordingly, the World Health Organization (WHO) has designated CRAB as a pathogen that poses a major threat to human health and that should be urgently targeted by new antibiotics (WHO, 2017).

A key step in containing CRAB is the rational use of antibiotics based on accurate diagnosis of infection. However, the challenge of differentiating CRAB colonization and infection could lead clinicians to prescribe excessive broad-spectrum antibiotics, potentially promoting the occurrence of drug-resistant bacteria and their spread in hospitals. Therefore, clinical infection control requires reliable methods for distinguishing CRAB colonization from CRAB infection. Reduction in lower respiratory tract (LRT) microbiota diversity or elevated abundance of certain strains may lead to infections (Dickson et al., 2014; Kelly et al., 2016). For example, LRT microbiota diversity was lower in 13 infected patients than in healthy control patients, and pathogenic bacteria in four subjects was consistent with the dominant microbiota identified by 16S rRNA analysis (Kelly et al., 2016). Through 16S rRNA analysis of 263 samples, Emonet et al. (2019) found that the low relative abundance of species in oropharyngeal secretions during intubation was strongly associated with subsequent VAP.

However, many aspects of the relationship between respiratory microecology and infection remain unknown, and current research is focused mainly on chronic lung diseases (Faner et al., 2017; Budden et al., 2019). Budden et al. (2019) reviewed recent advances in understanding the composition of the lung microbiome and found that bacteria, viruses, and fungi from the respiratory tract produce structural ligands and metabolites that interact with the host and alter the development and progression of chronic respiratory diseases. Hakansson et al. (2018) suggested that a complex interplay between the host, environment, and properties of the colonizing microorganisms determines disease development and severity. Moreover, Roquilly et al. (2019) proposed that the diversity of the microbiome and mucosal immunity are associated with hospital acquired pneumonia (HAP). Metagenomic analysis of microbiome structure and function will aid in understanding the pathogenesis and regulatory networks of AB during infection (Faner et al., 2017).

This is the first study to analyse and compare the characteristics of LRT microbiota of CRAB-negative, CRAB-colonized, and CRAB-infected patients using 16S rRNA, metagenomics, and whole-genome sequencing (WGS). We investigated whether VAP patients are associated with unique LRT microbiota to explore the pathogenesis of VAP at the level of the microbiota and provide an improved basis for clinical decision-making.

## Materials and Methods

### Study Design and Patient Enrolment

This prospective study was conducted from July 2018 to December 2019 in the adult ICUs of the First Affiliated Hospital, College of Medicine of Zhejiang University, China. The patient inclusion criteria were as follows: (1) patient was mechanically ventilated and hospitalized in the ICU; (2) Acute Physiology and Chronic Health Evaluation (APACHE) II score was greater than 12; and (3) collection of endotracheal deep aspirate (ETA) specimens was possible. Exclusion criteria were as follows: (1) age < 18 years; (2) comorbidity of chronic lung disease or lung cancer; (3) hospital stay < 24 h; and (4) co-infection with other bacteria. VAP was defined by the criteria of the Centers for Disease Control and Prevention (CDC) of the United States based on clinical, laboratory, radiological, and microbiological data (Horan et al., 2008). Patients meeting VAP criteria with positive culture for CRAB ETA were assigned to the CRAB VAP group (CRAB-I). Respiratory tract CRAB colonization (CRAB-C) was defined as CRAB-positive culture from ETA without VAP. Control patients (CRAB-N) were patients with neither CRAB VAP nor CRAB colonization. Approval was obtained from the ethical board of the hospital (reference number: 2016-458-1).

### Patient Sampling and Bacterial Isolation

The aspirate samples were transported to the microbiology laboratory within 2 h. ETAs were inoculated with a calibrated loop (0.001 ml). A gram-stained smear was prepared for all specimens and examined microscopically. If the number of squamous epithelial cells is less than 10/ each low power field, it is judged as qualified. MacConkey agar (Oxoid, United Kingdom) and incubated aerobically at 37°C overnight. *Acinetobacter baumannii* was identified by using biochemical methods and matrix-assisted laser desorption-time of flight mass spectrometry (MALDI-TOF MS) (Bruker, Bremen, Germany). The confirmed *A*. *baumannii* from clinical and sputum samples were stored at −80°C for further study. The resistance of *A*. *baumannii* to antibiotics, including Piperacillin/tazobactam (TZP), Cefoperazone/sulbactam (CSL), Ceftazidime (CAZ), Ceftriaxone (CRO), Cefepime (FEP), Ciprofloxacin (CIP), Levofloxacin (LVX), Imipenem (IPM), Meropenem (MEM), Trimethoprim/sulfamethoxazole (TMX/SXT), Amikacin (AMK), and Gentamicin (GEN), was analyzed according to the recommendation of Clinical and Laboratory Standards (CLSI, 2018). Resistance to Tigecycline (TGC) and Polymyxin (POL) was examined by the broth microdilution method and evaluated according to the European Committee on Antimicrobial Susceptibility testing (2016). *Escherichia coli* ATCC25922 was used as a quality control strain. CRAB was defined as AB strains that were non-susceptible to imipenem or meropenem. For CRAB-C group, we selected samples and strains cultured for the first time for metagenomics and whole-genome sequencing. If the patients were in CRAB-I group, we selected the samples of the latest infection state and the cultured strains for sequencing.

### Clinical Data

Clinical data including demographic variables, length of ICU stay, length of hospital stay, comorbidities, previous invasive procedures (central line insertion, intubation, continuous renal replacement therapy, and surgery under general an aesthesia), as well as lengths and types of antibiotic treatments and severity of illness [Acute Physiology and Chronic Health Evaluation (APACHE II)], were recorded (LeGall et al., 1986). Data on the levels of serum markers for liver and renal function [e.g., bilirubin, alanine transaminase (ALT), aspartate transaminase (AST), gamma glutamyl transpeptidase (γ-GT), urea, and creatinine] as well as those of blood biomarkers of infection other than core temperature [e.g., white blood cell (WBC) and CRP] were also collected.

### 16S rRNA Amplicon and Data Analysis

Total genomic DNA was extracted from samples using the cetyl trimethylammonium bromide (CTAB) method. We performed PCR amplification and pooled purified amplicons and then carried out the paired-end sequencing on an Illumina NovaSeq PE250 platform. After demultiplexing and trimming of the barcode and primer sequence using FLASH (V1.2.7) (Magoč and Salzberg, 2011), the paired-end raw read data of each sample was acquired. Subsequently, quality control was carried out using Qiime (Caporaso et al., 2010; Bokulich et al., 2013) and effective tags were obtained for analysis. OTUs, clustered with a 97% similarity cut-off using Usearch (Version 7.0) (Edgar et al., 2011), were taxonomically annotated using Mothur and SILVA database (Quast et al., 2013). MUSCLE (Version 3.8.31) software was used for rapid multiple sequence alignment and the phylogenetic analysis for all OTU representative sequences (Edgar, 2004). We then constructed the abundance matrices at the levels of phylum, class, order, family, and genus were constructed for each sample. Alpha diversity (Shannon and Simpson index within a sample) and Beta diversity (Bray–Curtis dissimilarity matrix across samples) were calculated using the *phyloseq* R package (version 1.32.0). PCoA and NMDS analysis based on Bray–Curtis dissimilarity was performed using the Vegan R package (version 2.5.6) and visualized using the ggplot2 R package (version 3.3.2). Analysis of similarity (ANOSIM) was used to test whether there is significant. We also calculated the co-occurrence relationships between bacteria using Python-based SparCC tool with SparCC correlation method and visualized using Cytoscape (Version 3.8.0) (Csardi and Nepusz, 2006). Sample names and the corresponding NCBI accession numbers are listed in Supplementary Table 1.

### Metagenomics Sequencing and Analysis

Following fragmentation of microbial DNA, metagenomic sequencing was performed on an Illumina NovaSeq 6000. After paired-end Illumina sequencing, we employed a previously reported bioinformatics pipeline to detect and profile the airway microbiome (Dickson et al., 2014; Kelly et al., 2016; Faner et al., 2017; Emonet et al., 2019). The low-quality sequences were filtered out or trimmed using PRINSEQ-lite (Version 0.19.3). And then *de novo* assemblies were generated using SPAdes genome assembler (Version 3.11.1) (Nurk et al., 2017) and coding sequences were predicted using MetaGeneMark (Version 3.38). Taxonomy assignments of both the clean reads and coding sequences were performed by Kaiju classifier (Version 1.7.2) (Menzel et al., 2016) with the National Center for Biotechnology Information Refseq database. We also utilized tool StrainPhlAn (Truong et al., 2017) to detected the multiple CRAB strains in ETA metagenomics samples. The functions of coding sequences were obtained using DIAMOND software (Version 0.9.30) with Kyoto Encyclopedia of Gene and Genomes (KEGG) database (Buchfink et al., 2015; Kanehisa et al., 2017). Furthermore, virulence genes in metagenomics sequences were identified by comparing the coding sequences against the Virulence Factor Database using DIAMOND software (Chen et al., 2016). The correlation coefficient between bacteria and virulence genes was generated using Python-based SparCC tool with SparCC correlation method. We performed multivariate linear regressions with feature selection, using the lasso penalized maximum likelihood technique in the “glmnet” R package (Version 4.0.2). Sample names and the corresponding NCBI accession numbers are listed in Supplementary Table 1.

### Whole-Genome Sequencing and Analysis

The genomic DNA of 46 isolates was extracted using a Qiagen DNA purification kit (Qiagen, Hilden, Germany) and sequenced on an Illumina HiSeq 4000-PE150. For each isolate, *de novo* assembly of reads was performed using SPAdes (version 3.11) genome assembler. CheckM was used to check genome completeness and possible contamination into the genome (Parks et al., 2015). We annotated the assemblies using Prokka (version 1.14.6) (Tatusova et al., 2016). GFF format files produced by Prokka were subjected to Roary (version 3.11.2) (Page et al., 2015) to construct core genome. Meanwhile, we analyzed whether there were multiple CRAB ST clones in one ETA sample using the raw reads through MetaMLST (Zolfo et al., 2017). A maximum-likelihood phylogenetic tree was constructed using RaxML software (version 8.2.12) with 1,000 bootstraps replicates (Price et al., 2010). Average nucleotide identity (ANI) was calculated by using a pyani (version 0.2.10). ANI values above 95% between genomes of these isolates denote the same source (Jain et al., 2018). We then conducted MLST analysis according to the Institute Pasteur scheme (MLST-IP) and Oxford Database (MLST-OD) (Gaiarsa et al., 2019). Clonal complexes (CCs) were assigned by eBURST and were defined as single locus (Hall, 2013). CCs were named according to the number of the predicted founder ST. Furthermore, we detected Antimicrobial resistance genes (ARG) and *AB* virulence genes by comparing genome assemblies against the ResFinder antibiotic resistance gene database using the Abricate software (version 0.8) and against the Virulence Factor Database using the DIAMOND software (version 0.9.30), respectively (Seemann, 2017). We also determined the capsular polysaccharide (KL) and lipooligosaccharide outer core (OCL) synthesis of the *A*. *baumannii* using Kaptive software (version 0.5.1) (Wyres et al., 2020). Strain names and the corresponding NCBI accession numbers are listed in Supplementary Table 1.

### Statistical Analysis

The Linear Discriminant Analysis Effect Size (LEfSe) program was used to identify bacterial taxa and virulence genes that were differently abundant between sample types (Segata et al., 2011). Normally distributed continuous variables were represented as means ± standard deviation (SD) and compared using Student’s *t*-test, whereas non-normally distributed continuous variables were represented as median and interquartile range (IQR) and compared using Mann–Whitney *U*-tests. Categorical variables were compared by the χ^2^ test or two-tailed Fisher’s exact test, as appropriate.

### Accession Numbers

All sequencing data during the current study are available in the Sequence Read Archive (SRA). Metagenomics data and the 16S rRNA gene data are under BioProject PRJNA 681291, and the WGS data under BioProject PRJNA 679997.

## Results

### Characteristics of the Study Population

**FIGURE 1:**
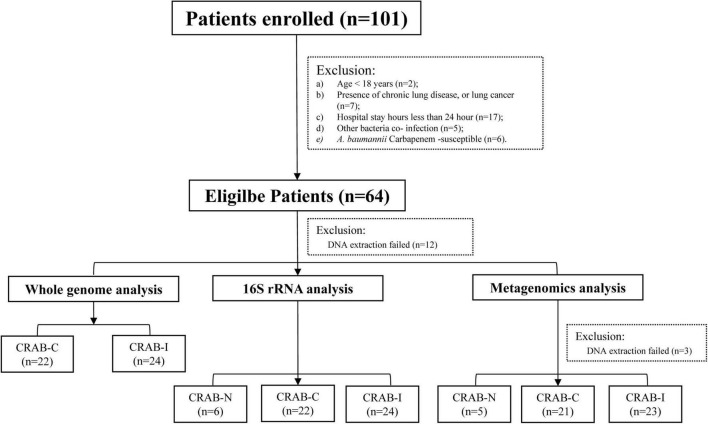
Flowchart of the patient enrollment process.

A total of 101 patients were screened. According to the inclusion criteria, 64 patients were enrolled, and 52 patients completed the entire study protocol (Figure 1): 24 with CRAB-I, 22 with CRAB-C, and six with CRAB-N. The CRAB-I and CRAB-C patients did not differ in terms of age, sex, or severity indices (APACHE II scores), but C-reactive protein (CRP) and 30 day mortality were higher in CRAB-I (Supplementary Table 2). There was no significant difference in the prior antibiotic therapy between CRAB-C and CRAB-I. All CRAB isolates were highly resistant to all antibiotics except amikacin, polymyxin, and tetracycline (Supplementary Figure 1).

### Comparison of Microbiota of Endotracheal Deep Aspirate

#### Overview

16S rRNA sequencing from a total of 52 ETA specimens revealed that the average Operation taxonomic units (OTU) numbers for CRAB-N, CRAB-C, and CRAB-I were 1,427, 2,422, and 2,248, respectively. Rarefaction curves of numbers of observed OTUs per sample and group indicated that almost all OTUs present in each group were detected (Supplementary Figure 2). Three samples failed to sequence. The LRT microbiome was examined in the remaining 49 ETA specimens by shotgun metagenomic sequencing (Supplementary Table 1). The 16S rRNA sequencing data identified 597 genera, of which 249 (41.2%) were also identified in the metagenomic data (Supplementary Figures 3, 4). All of the alpha diversity indices in the metagenomic analysis were higher than those in the 16S amplicon analysis (Supplementary Figure 5). Interestingly, only single ST was found in each metagenomics sample, which was the same ST as that was identified in the corresponding WGS data.

#### Carbapenem-Resistant *Acinetobacter baumannii*-Positive Patients Had Reduced Microbiota Diversity

**FIGURE 2:**
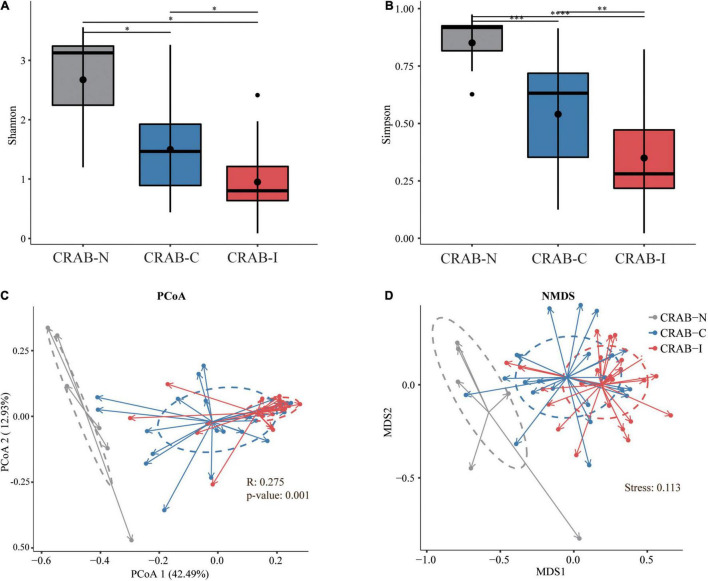
Phylogenetic diversity comparison of lower respiratory tract (LRT) microbiota in LRT microbiota of patients with neither VAP nor CRAB LRT colonization (CRAB-N), LRT microbiota of patients with CRAB colonization but without VAP (CRAB-C), and LRT microbiota of patients who developed CRAB VAP (CRAB-I) patients. **(A)** Box plots depict microbiota diversity differences according to the Shannon index among three group. **(B)** Box plots depict microbiota diversity differences according to the Gini Simpson index among three group. The upper and lower ranges of the box represent the 75 and 25% quartiles, respectively. **(C)** The X-axis shows PCo 1 and the Y-axis PCo 2 form Bray-Curtis distances for β-diversity. The changes per patient are visualized in as dashed lines dark red for CRAB-I, in blue for CRAB-C and in gray for CRAB-N. Logistic regression analysis showed that the change of PCo1 was significantly lower in patients who developed pneumonia (*P* = 0.001). **(D)** The X-axis shows MDS 1 and the Y-axis MDS 2 form Bray-Curtis distances for β-diversity (Stress = 0.113). The changes per patient are visualized in as dashed lines dark red for CRAB-I, in blue for CRAB-C and in gray for CRAB-N. Significant differences are indicated by *P < 0.05, ^**^P < 0.01, ^***^P < 0.001, and ^****^P < 0.0001.

Relative to CRAB-N, the diversity of pulmonary microbiota in the CRAB-C group was significantly reduced, and a further reduction was observed in the CRAB-I group (Shannon index in CRAB-N, -C, and -I: 4.80 ± 1.47, 2.73 ± 1.24, and 1.79 ± 0.95; Simpson index: 0.90 ± 0.08, 0.60 ± 0.23, and 0.40 ± 0.20, respectively; *P* < 0.05 for all alfa-diversity index; Figures 2A,B). Moreover, principal co-ordinate analysis (PCoA) and non-metric multidimensional scaling (NMDS) analysis of the Bray–Curtis dissimilarity metric revealed that the composition of the microbiota between the three groups were quite different (*P* = 0.001 and Stress = 0.113; respectively; Figures 2C,D). Analysis of similarity (ANOSIM) comparative analysis revealed that the differences between the three groups were higher than those within each group, indicating that the microbial community structure of the LRT microbiota in the three groups was significantly distinct (Supplementary Table 3).

#### Bacterial Taxonomic Characters in the Three Groups

**FIGURE 3:**
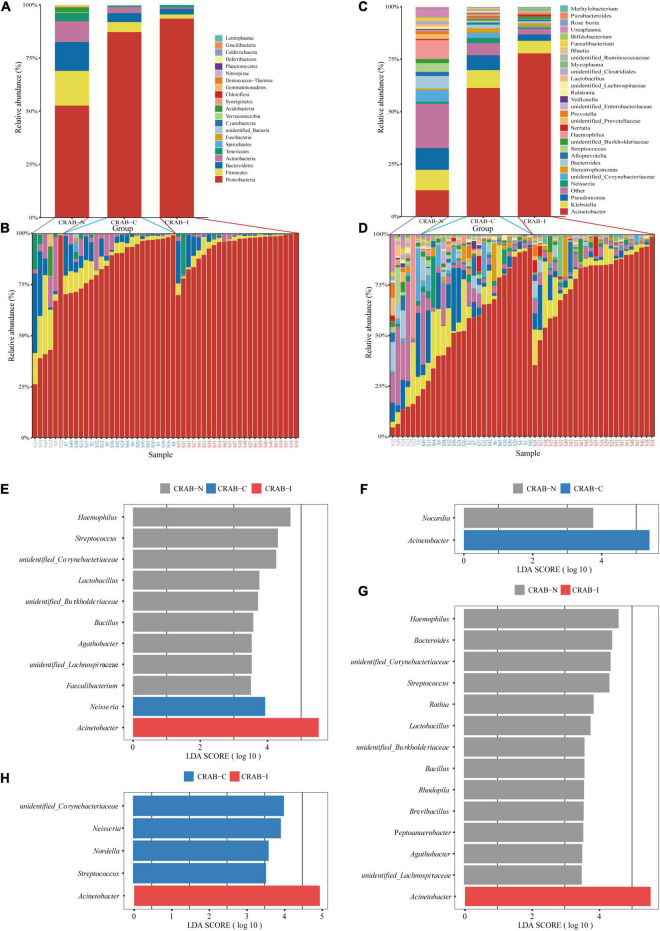
Comparison of phylum and genus of the LRT microbiota in the three patient groups and linear discriminant analysis effect size (LEfSe) analysis of the microbiota at genus level. **(A)** Comparison of the average abundance of each bacterial phylum in each group, respectively. **(B)** Comparison of the average abundance of bacterial phylum in each patient, respectively. **(C)** Comparison of the average abundance of each bacterial genus in each group, respectively. **(D)** Comparison of the average abundance of bacterial genus in each patient, respectively. The changes per patient are visualized in as dashed lines dark red for CRAB-I, in blue for CRAB-C and in gray for CRAB-N. **(E)** LDA scores indicated significant differences in the microbiota among the CRAB-N (gray), CRAB-C (blue), and CRAB-I patients (red). **(F)** LDA scores indicated significant differences in the microbiota between the CRAB-N (gray) and CRAB-C patients (blue). **(G)** LDA scores indicate significant differences in the microbiota between the CRAB-I patients (red) and CRAB-N controls (gray). **(H)** LDA scores indicate significant differences in the microbiota between the CRAB-I (red) and CRAB-C patients (blue). LDA scores > 3.5. The subject group is indicated by the color key at the top right corner.

To obtain a global view of LRT microbiota in the study subjects, we compared the taxa at the phylum and genus levels between the three groups by 16S rRNA amplicon analysis. Overall, Proteobacteria (43.57%), Firmicutes (2.58%), and Bacteroidetes (2.42%) were the dominant phyla in the 52 ETA specimens (Figure 3A). The relative abundance of Proteobacteria was significantly higher in the CRAB-I and CRAB-C groups than in the CRAB-N group (91.77 vs. 84.50 vs. 49.25%, *P* < 0.05), and Firmicutes exhibited the opposite pattern (2.23 vs. 4.38 vs. 15.67%, *P* < 0.05; Figure 3B). At the genus level, the bacterial composition of the CRAB-positive groups differed from that of the negative group. *Acinetobacter* was the predominant genus in CRAB-I and CRAB-C patients (76.19 vs. 59.14%), followed by *Klebsiella* (5.80 vs. 8.01%) and *Pseudomonas* (2.84 vs. 6.88%). By contrast, in the CRAB-N group, the top three genera were *Acinetobacter* (11.24%), *Haemophilus* (8.67%), and *Pseudomonas* (9.68%) (Figures 3C,D).

The Linear Discriminant Analysis Effect Size (LEfSe) comparison identified 11 significant biomarker genera (Figure 3E) with discrimination value in the three groups. Further pairwise comparison revealed two significant biomarker genera (*Acinetobacter* and *Nocardia*) between the CRAB-C and CRAB-I groups (Figure 3F), and the CRAB-I group had 14 significant biomarker genera with the CRAB-N group: *Acinetobacter* was enriched in the CRAB-I group and 13 other genera, including *Haemophilus*, *Bacteroides*, and *Streptococcus*, were enriched in the CRAB-N group (Figure 3G). In addition, five key genera (*Acinetobacter*, *unidentified_Corynebacteriacea*, *Nesseria*, *Nordella*, and *Streptococcus*) differentiated between the CRAB-C and CRAB-I groups (Figure 3H).

#### Identifying Potential Microbial Interactions by Correlation Network Analysis

**FIGURE 4:**
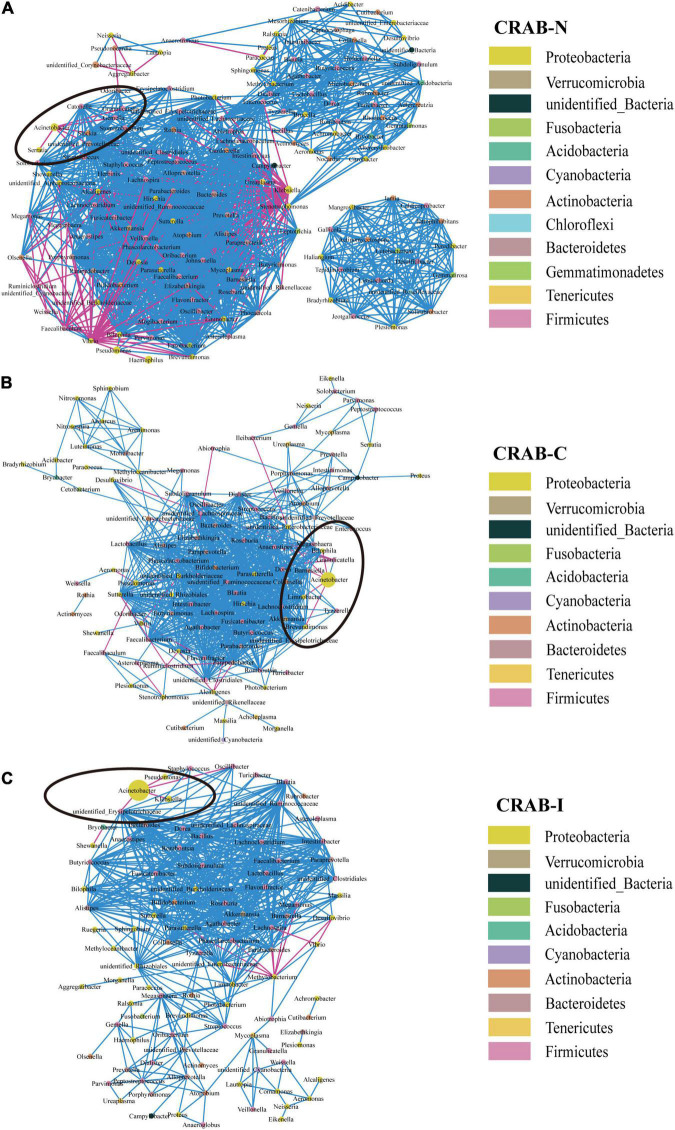
Co-occurring network of microbial communities in LRT samples from CRAB-N **(A)**, CRAB-C **(B)**, and CRAB-I patients **(C)**. A co-occurring network containing strong (ρ > 0.6) and significant (FDR-adjusted *P* < 0.05) correlations was represented. Each node represents a genusa and the nodes are colored by phylum. The size of each node is proportional to the number of connections. The thickness of each edge is proportional to the ρ. Light blue lines represent negative correlations, and red lines represent positive correlations.

Overall, the microbial co-occurrence network constructed from CRAB-I had a lower complexity index than those of CRAB-C and CRAB- N at the genus level (4,239.37 vs. 4,257.25 vs. 7,459.00) (Figure 4). The total number of negative microbial interactions, as indicated by the number of edges between the nodes, was highest in CRAB-N, moderate in CRAB-C, and lowest in CRAB-I (CRAB-N: *n* = 2,356, 2,189 positive and 167 negative; CRAB-C: *n* = 1,436, 1,411 positive and 25 negative; CRAB-I: *n* = 1,533, 1,516 positive and 17 negative). Notably, in the CRAB-I group, *Acinetobacter* was abundant and significantly negatively correlated with four genera (*Klebsiella*, *Pseudomonas*, *unidentified_Erysipelotrichaceae*, and *Oscillibacter)*, whereas in the CRAB-C group, it was negatively correlated with five other members of the microbiota (*Limnobacter*, *Brevundimonas*, *Dialister*, *Barnesiella*, and *Bilophila*), and in the CRAB-N group, it had six negative connections. Thus, the number of negative interactions decreased much more between CRAB-N and CRAB-I than the number of positive interactions.

### Comparison of Functional Profiles of Microbiota

**FIGURE 5:**
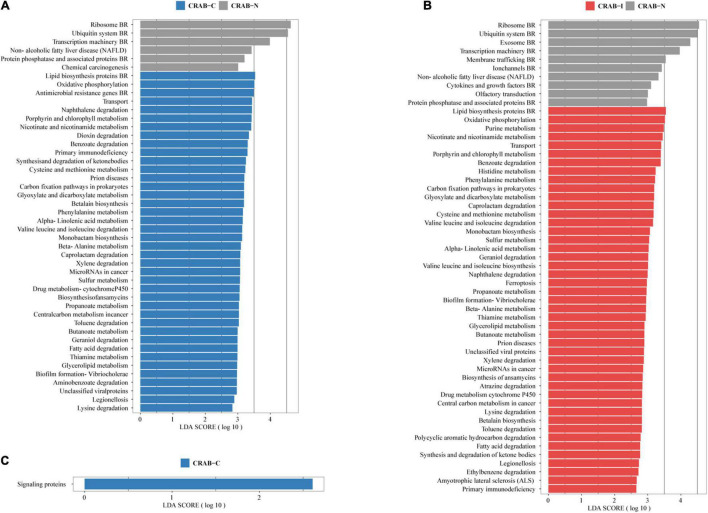
Functionally distinct and LEfSe analysis based on KEGG pathway. **(A)** Linear discriminant analysis (LDA) scores indicate significant differences in the microbiota between the CRAB-C patients (blue) and CRAB-N controls (gray); **(B)** LDA scores indicate significant differences in the microbiota between the CRAB-I patients (red) and CRAB-N controls (gray); **(C)** LDA scores indicate significant differences in the microbiota between the CRAB-C patients (blue) and CRAB-I patients (red). LDA scores > 2.5. CRAB, carbapenem-resistant *Acinetobacter baumannii*; LRT, lower respiratory tract; VAP, ventilator associated pneumonia; CRAB-N, LRT microbiota of patients with neither VAP nor CRAB LRT colonization; CRAB-C, LRT microbiota of patients with CRAB colonization but without VAP; CRAB-I, LRT microbiota of patients who developed CRAB VAP.

The Linear Discriminant Analysis Effect Size analysis using a logarithmic LDA score cut-off of 2.5 identified 46 and 55 different pathways in CRAB-C and CRAB-I, respectively, relative to CRAB-N, of which 40 and 45, respectively, increased. These pathways included fatty acid degradation, oxidative phosphorylation, nicotinate and nicotinamide metabolism, transport, porphyrin and chlorophyll metabolism, benzoate degradation, and biofilm formation in *Vibrio cholerae* (Figures 5A,B). Moreover, signaling proteins (KEGG pathway ko99995, Figure 5C) were more active in CRAB-C than in CRAB-I.

### Comparison of Virulence Factors in the Microbiome

**FIGURE 6:**
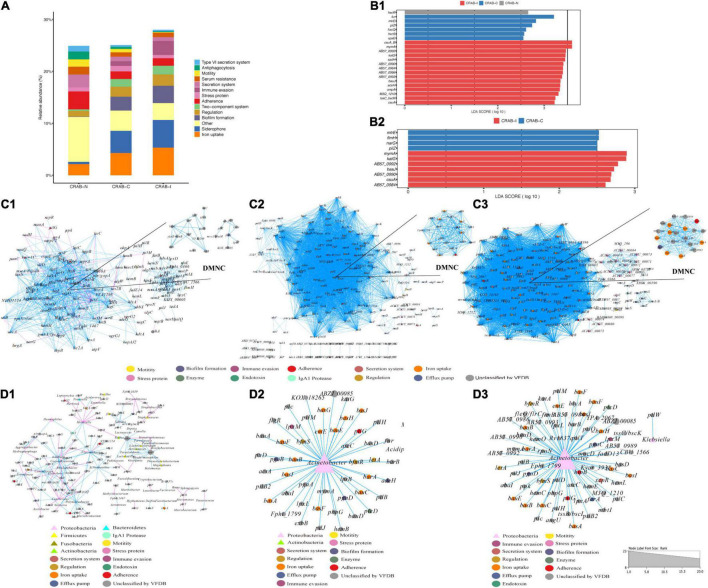
Comparison of virulence factors and Network analysis. **(A)** Summary of the top 10 relative abundances of function group of virulence genomes in each group; **(B)** LEfSe and LDA scores indicated significant differences in the virulence genes among groups. **(B1)** Represented differences among the CRAB-N, CRAB-C, and CRAB-I patients; **(B2)** Represented differences between the CRAB-C and CRAB-I patients. **(C)** Co-occurring network of virulence genomes communities in LRT samples from three patient groups (**C1**: CRAB-N, **C2**: CRAB-C, **C3**: CRAB-I). The nodes are colored by the function of virulence genomes, the upper right corner shows DMNC; **(D)** Co-occurring network of virulence genomes and microbiota communities in LRT samples from three patient groups based on correlation analysis (**D1**: CRAB-N, **D2**: CRAB-C, **D3**: CRAB-I). The nodes are shaped by virulence genomes and bacteria, sized by the relative abundances and colored by the function of virulence genomes or phylum. The connections in the network represent a strong (*r* > 0.8) and significant (*q* < 0.05) correlations. The size of each node is proportional to the number of connections. Light blue lines represent positive correlations, and red lines represent negative correlations.

**FIGURE 7:**
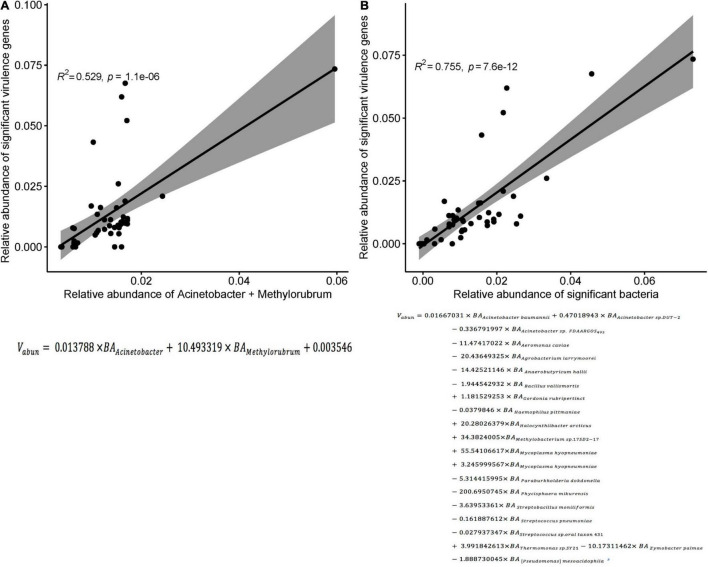
Correlation between the sum of the relative abundances of genus or species and the significant virulence genes density. **(A)** Correlation between the sum of the relative abundances of Acinetobacter and Methylorubrum and the significant virulence genes density in each sample (R^2^ = 0.529, *p* < 0.0001; Pearson’s correlation). **(B)** Correlation between the sum of the relative abundances of species and the significant virulence genes density in each sample (R^2^ = 0.755, *p* < 0.0001; Pearson’s correlation). *V*_*abun*_, Sum of abundance of significant virulence genes; *BA*_*i*_: abundance of bacteria *i*.

In the three groups of patients, we detected a total of 1,628 virulence genes, divided into 69 functional groups. The top 10 most abundant functional virulence groups in each group accounted for 26.5% of all factors, and the composition of the “toxicity” functional group differed between the three groups of patients (Figure 6A). These factors tended to be involved in iron uptake, siderophore biosynthesis, immune evasion, and biofilm formation. Ninety-five virulence genes (relative abundance > 0.1% for each) differed in abundance between CRAB-N and CRAB-C, and 105 differed between CRAB-N and CRAB-I (Supplementary Table 4). Eleven genes, including AB57_ 0984, AB57_ 0990, AB57_ 0992, and mymA, were more abundant in CRAB-I than in CRAB-C (Figure 6B and Supplementary Table 4). Virulence gene networks constructed from CRAB-I had a higher complexity index than those from CRAB-C and CRAB-N patients (56,206.99 vs. 44,722.75 vs. 11,052.12) (Figure 6C). More virulence genes associated with *Acinetobacter* were detected in CRAB-I than in CRAB-C and CRAB-N, with functions including immune evasion, iron uptake, and VFDB-unclassified (Figure 6D). Consistent with this association, the levels of VFs and the relative abundances of genera (*Acinetobacter* and *Methylorubrum*) or species exhibited a strong and significant positive correlation (R2 = 0.529, *P* = 1.1e-06; R2 = 0.755, *P* = 7.6e-12, Pearson’s correlation; Figures 7A,B), indicating that differences in the abundance of VFs were driven by differences in the species present in each group of patients.

### Whole-Genome Analysis of Carbapenem-Resistant *Acinetobacter baumannii*

**FIGURE 8:**
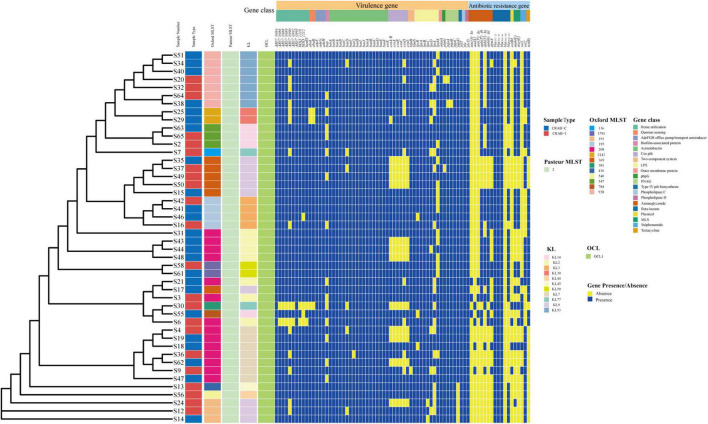
Molecular type, resistant and virulence genes analysis of *Acinetobacter baumannii* isolates.

We calculated quality of microbial genomes and found the completeness was 99.85% ± 0.27% and the contamination was 0.96 ± 0.43. The average nucleotide identity (ANI) of the 46 CRAB strains was >95%, indicating that they belonged to the same species (Supplementary Table 1 and Supplementary Figure 6A). MLST of 46 CRAB was dominated by ST208 (30.43%), followed by ST938 (15.22%). eBURST analysis revealed that seven ST types (87.5%) clustered in the same clonal complexes (CCs) (CC92) (Supplementary Figure 6B). The KL types were mainly KL9, KL2, KL93, and KL7, and all strains belonged to the OCL1 type (Figure 8). We observed no statistically significant difference in ST or KL type between the CRAB-I and CRAB-C groups (*P* = 0.478 and 0.444; respectively).

All CRAB isolates harbored *bla*_*ADC–25*_, *bla*_*OXA–23*_, and *bla*_*OXA–66*_. All isolates harbored more than one oxacillinase gene, and 28 (60.8%) harbored the class A β-lactamase gene *bla*_*TEM*_. The number of resistance genes did not differ significantly between the two groups (Figure 8). Annotation and analysis of virulence genes showed that strains from CRAB-I had more virulence genes than those from CRAB-C (Figure 8), and chi-square tests (Table 1) revealed that AB57_0990, Lpxl, and ABZJ_00085 were more abundant in CRAB-I, consistent with the metagenomics analysis (Figure 6B).

**TABLE 1:** The Virulence genes with significant difference between infection and colonization groups of carbapenem-resistant *Acinetobacter baumannii*.

VFs	Function	CRAB-C (*n* = 22)	CRAB-I (*n* = 24)	Chi states	*P* values
*lpxL*	LPS	8 (36.36)	19 (79.17)	8.674	0.003
*ABZJ_00085*	Capsule	7 (31.82)	16 (66.67)	5.576	0.018
*AB57_0990*	Heme utilization	11 (50)	19 (79.17)	4.305	0.038

*VFs, virulence factors; CRAB, carbapenem-resistant Acinetobacter baumannii; VAP, ventilator associated pneumonia; CRAB-C, positive genes of patients with CRAB colonization but without VAP; CRAB-I, positive genes of CRAB VAP patients.*

## Discussion

Carbapenem-resistant *Acinetobacter baumannii* has a high clinical prevalence and is a common pathogen in VAP, but a positive ETA culture alone cannot effectively distinguish between bacterial colonization and infection, representing a longstanding clinical challenge in the management of severely ill patients. Therefore, we investigated the difference in LRT microecology between infected and colonized patients using multi-genomics methods, with the goal of clarifying the clinical management of CRAB infection.

In recent years, several studies have shown that changes in the LRT microbiome are related to the occurrence of lung diseases, but few studies have examined the relationship between respiratory microbiota and infection, and most of those focused on pulmonary tuberculosis and pulmonary fibrosis (Krishna et al., 2016; Emonet et al., 2019). In this study, the 16S rRNA analysis of 52 patients revealed that the α and β diversity of the LRT microbiome was significantly lower in CRAB-I patients than in CRAB-C and CRAB-N patients (Figure 2). The ETA microbiota in the CRAB-N group consisted mainly of Proteobacteria and *Haemophilus*, consistent with a previous report (Dickson et al., 2017), and was more diverse than in the CRAB-C and CRAB-I groups. The microbiota of the latter two groups were mainly Proteobacteria and *Acinetobacter*, and the abundance of *Acinetobacter* in CRAB-I was as high as 76.19% (Figure 3). Further LEfSe analysis (Figure 3H) revealed that, in comparison with CRAB-C patients (who were enriched in *unidentified_Corynebacteriacea*, *Nesseria*, *Nordella*, and *Streptococcus*), CRAB-I patients had a higher abundance of *Acinetobacter*. The relative abundance of *Acinetobacter* increased in the order CRAB-N, CRAB-C, and CRAB-I; this trend was confirmed by Woo et al. (2020). Together, these results indicated that a dynamic evolution of pulmonary microbiota, including a decline in diversity and enrichment of *Acinetobacter*, occurs prior to the onset of CRAB VAP (Dickson et al., 2016; Zakharkina et al., 2017).

Network analysis revealed that the connections between bacteria were most abundant in CRAB-N, less abundant in CRAB-C, and least abundant in CRAB-I; in parallel, the number of genera negatively associated with *Acinetobacter* also decreased (6, 5, and 4 negative connections in CRAB-N, CRAB-C, and CRAB-I, respectively). In CRAB-I, only four genera (*Klebsiella*, *Pseudomonas*, *unidentified_Erysipelotrichaceae*, and *Oscillibacter*) were negatively correlated with *Acinetobacter* (Figure 4). Zakharkina et al. (2017) found that *Acinetobacter*, *Pseudomonas*, *Staphylococcus*, and *Burkholderia* were negatively correlated with the development of VAP; Wouter et al. (de Steenhuijsen Piters et al., 2016) found that an increase in the abundance of *Lactobacillus* and *Rothia* strains was negatively correlated with the specific microbial infection of VAP patients. These findings suggest that disturbance of the respiratory microbiota relieves negative inhibition of CRAB and is therefore likely to promote infection of the host. However, this idea requires further validation.

Functional metagenomic studies of the respiratory tract microbiome are also valuable for detecting bacterial pathogenesis. Mice infected with *Streptococcus pneumoniae* and *Haemophilus influenzae* could cause pulmonary inflammatory responses by activating the MAPK signal pathway (Weiser et al., 2018). In this study, KEGG functional analysis revealed that genes involved in 40 and 45 metabolic pathways (including oxidative phosphorylation, phenylalanine metabolism, fatty acid degradation) were more abundant in the CRAB-C and CRAB-I groups, respectively, than in the CRAB-N group; moreover, signaling protein pathways were more active in CRAB-C patients than in CRAB-I patients (Figure 5). A previous review described how assessment of microbial function using metagenomics, metatranscriptomics, and metabolomics has identified metabolites produced by respiratory microbiota (especially fatty acids, sugars, and amino acids) that can influence host immunity (Budden et al., 2019). This also indicated that the change in bacterial pathogenicity from CRAB-N to CRAB-I may be associated with more active metabolism; this possibility is worthy of further study.

During progression from colonization to infection, bacterial invasiveness and toxicity play a key role. Metagenomics analysis revealed that four major virulence gene clusters (iron uptake, siderophore, immune evasion, and biofilm formation) increased in abundance from the CRAB-N to the CRAB-I group (Figure 6A). The number of virulence genes annotated and networks constructed was significantly higher in the CRAB-I group in the CRAB-C and CRAB-N groups (Figure 6C). Wilcoxon tests showed that the abundance of virulence genes related to heme utilization, such as AB57_ 0984, AB57_ 0990, AB57_ 0992, and mymA, was higher in the CRAB-I group than in the CRAB-C group (Supplementary Table 4 and Figure 6B). AB57_ 0984, a LysR family transcription regulator, is linked to elevated invasiveness (Gebhardt et al., 2020). AB57_ 0990 (a member of the TonB family) and the TonB system play key roles in the pathogenicity of AB (Runci et al., 2019). Network diagram and fitted curve analysis confirmed that these virulence genes were associated with *Acinetobacter* (Figures 6D, 7). In addition, WGS of strains from CRAB-C and CRAB-I patients revealed that more virulence genes such as Lpxl, ABZJ_00085 and AB57_0990 were present in the infection group (Table 1 and Figure 8). The enrichment in virulence of CRAB indicated that enhancement of bacterial pathogenicity could be another key factor that promotes infection after perturbation of the microbiota.

In terms of patient clinical characteristics, we observed no significant differences in gender, age, or severity of disease at the time of enrolment between the three groups. The number of days of mechanical ventilation before collection was smaller in CRAB-N patients than in CRAB-positive patients, and mortality was higher in CRAB-I patients (Supplementary Table 2). ST208 was the main type (30.43%), followed by ST938 and ST195, and bacterial MLST distribution did not differ significantly between the colonization and infection group (Figure 8). All isolates were highly drug-resistant, and *bla*_*oxa–23*_ was the major determinant of resistance (Fu et al., 2010).

Our results indicate that to draw conclusions about the importance of microbiota evolution, it will be necessary to perform consecutive observations of individual patients, spanning the period from colonization to infection. In future studies, transcriptome and proteome analysis could be used to explore germ–host interactions and pathogenesis.

## Conclusion

By combining 16S rRNA amplicon analysis, metagenomics sequencing, and WGS, we characterized the respiratory tract microbiome of patients with CRAB VAP and explored the differences in microbiota between bacterial colonization and infection. Our results revealed that LRT microbiome dysbiosis, including declining diversity, the rise of *Acinetobacter* to dominance, weakening of the negative regulation of *Acinetobacter*, and significant enhancement of virulence, could promote the occurrence of infection. Thus, multi-genomics investigational methods could be used to develop new diagnostic measures for CRAB VAP.

## Data Availability Statement

The datasets presented in this study can be found in online repositories. The names of the repository/repositories and accession number(s) can be found below: https://www.ncbi.nlm.nih.gov/, PRJNA681291 https://www.ncbi.nlm.nih.gov/, PRJNA679997.

## Ethics Statement

The studies involving human participants were reviewed and approved by the ethical board of the Research Ethics Committee of the First Affiliated Hospital of Medicine, Zhejiang University in China (No. 2016-458-1). Written informed consent for participation was not required for this study in accordance with the national legislation and the institutional requirements. Written informed consent was not obtained from the individual(s) for the publication of any potentially identifiable images or data included in this article.

## Author Contributions

YX, HZ, and TX designed the study. QG, TX, ML, LG, XY, YL, and CW analyzed and interpreted the data. YZ, YW, SZ, XC, and XK collected the samples. PS, KZ, BZ, QL, and YC oversaw the field projects. QF provided clinical support. TX, QG, and YZ wrote the manuscript. All authors critically reviewed the manuscript and approved the submitted version.

## Conflict of Interest

The authors declare that the research was conducted in the absence of any commercial or financial relationships that could be construed as a potential conflict of interest.

## Publisher’s Note

All claims expressed in this article are solely those of the authors and do not necessarily represent those of their affiliated organizations, or those of the publisher, the editors and the reviewers. Any product that may be evaluated in this article, or claim that may be made by its manufacturer, is not guaranteed or endorsed by the publisher.

## Supplementary Material

Supplementary Material 1 Comparative respiratory tract microbiome between Carbapenem-resistant *Acinetobacter baumannii* colonization and ventilator associated pneumonia.

## Abbreviations

CRAB: carbapenem-resistant *Acinetobacter baumannii*
LRT: lower respiratory tract
VAP: ventilator associated pneumonia
CRAB-N: LRT microbiota of patients with neither VAP nor CRAB LRT colonization
CRAB-C: LRT microbiota of patients with CRAB colonization but without VAP
CRAB-I: LRT microbiota of patients who developed CRAB VAP
LefSe: linear discriminant analysis effect size
LDA: linear discriminant analysis
DMNC: density of maximum neighborhood component
VFs: virulence factors.
