# Corrigendum: Comparative respiratory tract microbiome between carbapenem-resistant *Acinetobacter baumannii* colonization and ventilator associated pneumonia

**DOI:** 10.3389/fmicb.2022.985295

**Published:** 2022-07-20

**Authors:** Tingting Xiao, Qian Guo, Yanzi Zhou, Ping Shen, Yuan Wang, Qiang Fang, Mo Li, Shuntian Zhang, Lihua Guo, Xiao Yu, Yulin Liao, Chunhui Wang, Xiaohui Chi, Xiaoyang Kong, Kai Zhou, Beiwen Zheng, Qixia Luo, Yunbo Chen, Huaiqiu Zhu, Yonghong Xiao

**Affiliations:** ^1^State Key Laboratory for Diagnosis and Treatment of Infectious Diseases, National Clinical Research Center for Infectious Diseases, National Medical Center for Infectious Diseases, Collaborative Innovation Center for Diagnosis and Treatment of Infectious Diseases, The First Affiliated Hospital, Zhejiang University School of Medicine, Hangzhou, China; ^2^State Key Laboratory for Turbulence and Complex Systems, Department of Biomedical Engineering, College of Future Technology and Center for Quantitative Biology, Peking University, Beijing, China; ^3^Department of Intensive Care Unit, The First Affiliated Hospital, Zhejiang University School of Medicine, Hangzhou, China; ^4^Shenzhen Institute of Respiratory Diseases, The First Affiliated Hospital (Shenzhen People's Hospital), Southern University of Science and Technology, Shenzhen, China

**Keywords:** carbapenem-resistant *Acinetobacter baumannii* (CRAB), ventilator-associated pneumonia (VAP), multi-genomics analysis, microbiome, virulence gene

In the published article, there was an error regarding the affiliation of “Yonghong Xiao.” Instead of having affiliation 2, they should have had affiliation 1.

The authors apologize for this error and state that this does not change the scientific conclusions of the article in any way. The original article has been updated.

## Publisher's note

All claims expressed in this article are solely those of the authors and do not necessarily represent those of their affiliated organizations, or those of the publisher, the editors and the reviewers. Any product that may be evaluated in this article, or claim that may be made by its manufacturer, is not guaranteed or endorsed by the publisher.

