# Supplementary material for: Comparative Respiratory Tract Microbiome Between Carbapenem-Resistant Acinetobacter baumannii Colonization and Ventilator Associated Pneumonia
Source: Front Microbiol. 2022 Mar 4;13:782210. doi: 10.3389/fmicb.2022.782210 (PMC8931608; doi:10.3389/fmicb.2022.782210)
Supplement: Supplementary Material 1 — Comparative respiratory tract microbiome between Carbapenem-resistant Acinetobacter baumannii colonization and ventilator associated pneumonia. [file Data_Sheet_1.ZIP › Frontiers Supplementary/Table S3.docx]

**Supplemental Table 3.** Anosim analyzes differences in community structure between groups.

| **Group** | **R-value** | **P-value** |
| --- | --- | --- |
| CRAB-I vs. CRAB-N | 0.9582 | 0.001 |
| CRAB-C vs. CRAB-N | 0.8018 | 0.001 |
| CRAB-C vs. CRAB-I | 0.1292 | 0.002 |

Abbreviations: CRAB, Carbapenem -resistant *Acinetobacter baumannii*; LRT, lower respiratory tract; VAP, Ventilator associated pneumonia; CRAB-N, LRT microbiota of patients with neither VAP nor CRAB LRT colonization; CRAB-C, LRT microbiota of patients with CRAB colonization but without VAP; CRAB-I, LRT microbiota of patients who developed CRAB VAP.
