# Supplementary material for: Comparative Respiratory Tract Microbiome Between Carbapenem-Resistant Acinetobacter baumannii Colonization and Ventilator Associated Pneumonia
Source: Front Microbiol. 2022 Mar 4;13:782210. doi: 10.3389/fmicb.2022.782210 (PMC8931608; doi:10.3389/fmicb.2022.782210)
Supplement: Supplementary Material 1 — Comparative respiratory tract microbiome between Carbapenem-resistant Acinetobacter baumannii colonization and ventilator associated pneumonia. [file Data_Sheet_1.ZIP › Frontiers Supplementary/Table S2∩╝êclean∩╝ë.docx]

**Table S2. Demographics and clinical parameters of patients enrolled in the study.**

|  |  |  | **CRAB-N(n=6)** | **CRAB-C(n=22)** | **CRAB-I(n=24)** | **P value ^e^** |
| --- | --- | --- | --- | --- | --- | --- |
| Demographic | | |  |  |  |  |
|  | male, n (%) | | 4(66.7) | 14(63.6) | 16(66.7) | 0.829 |
|  | Age, years, mean ± SD | | 51.8±15.1 | 58.5±16.9 | 60.7±14.0 | 0.631 |
|  | Total hospital stay, days, media (IQR) | | 37.5(12.3-81.3) | 26(16.5-58) | 27(15.3-41) | 0.628 |
|  | Prior hospital stay, days, media (IQR)^a^ | | 1.5 (1–4) | 10(3-16) | 7(4-10.8) | 0.408 |
| Mortality in 30-days | |  | 0 | 1(4.5) | 8(33.3) | 0.023 |
| Preexisting medical conditions | | | | |  |  |
|  | Pulmonary infection | | 0 | 2(9.1) | 6(25.0) | / |
|  | Heart disease | | 0 | 1(4.5) | 3(12.5) | / |
|  | Pancreatitis | | 1(16.7) | 2(9.1) | 1(4.2) | / |
|  | Intestinal disease | | 1(16.7) | 2(9.1) | 2(8.3) | / |
|  | Septic shock | | 0 | 0 | 3(12.5) | / |
|  | Cerebrovascular disease | | 3(50) | 5(22.7) | 6(25.0) | / |
|  | Brain trauma | | 0 | 3(13.6) | 1(4.2) | / |
|  | Multiple injuries | | 0 | 4(18.2) | 2(8.3) | / |
|  | Others | | 1(16.7) | 3(13.6) | 0 | / |
| Smoking history^a^ | |  | 2(33.3) | 8(36.4) | 9(37.5) | 0.936 |
| Alcoholic Drinking history^a^ | |  | 2(33.3) | 6(27.3) | 4(16.7) | 0.608 |
| Prior invasive procedure or devices, days (IQR)^b^ | | |  |  |  |  |
|  | Mechanical ventilation | | 1.5(1-4) | 10(3-15.3) | 7(4-9.8) | 0.372 |
| Prior receipt of antibiotics within 14 days^b^ | | |  |  |  |  |
|  | Cephalosporin | | 3(50) | 6(27.3) | 2(0.08) | 0.128 |
|  | Cefoperazone/Sulbactam | | 1(16.7) | 6(27.3) | 6(25.0) | >0.05 |
|  | Piperacillin/ Tazobactam | | 2(33.3) | 9(40.9) | 10(41.7) | >0.05 |
|  | Carbapenem | | 2(33.3) | 3(13.6) | 7(29.2) | 0.289 |
|  | Fluoroquinolone | | 1(16.7) | 0 | 3(12.5) | 0.235 |
|  | Tigecycline | | 0 | 2(9.1) | 6(25.0) | 0.247 |
|  | Polymyxins | | 0 | 1(4.5) | 4(16.7) | 0.345 |
|  | Anti-positive bacteria drugs | | 1(16.7) | 9(40.9) | 6(25.0) | 0.348 |
|  | Antifungal drugs | | 1(16.7) | 4(18.2) | 3(12.5) | 0.694 |
| Laboratory examination^c^ | | |  |  |  |  |
|  | WBC (10^9/L) | | 8.8(5.7-9.5) | 8.8(5.1-10.7) | 10.4(6.9-14.7) | 0.101 |
|  | CRP (mg/L) | | 20.9(15.5-69.86) | 33.3(19.0-62.2) | 101.4(38-158.2) | 0.017 |
| Severity of illness^c^ | | |  |  |  |  |
|  | APACHE II score (Mean ± SD) | | 13.0±5.2 | 13.3±10.6 | 16.5±8.5 | 0.265 |

Data are expressed as numbers (%) unless otherwise stated; Abbreviations: Abbreviations: CRAB, Carbapenem -resistant *Acinetobacter baumannii*; VAP, Ventilator associated pneumonia; CRAB-N, patients with neither VAP nor CRAB colonization; CRAB-C, patients with CRAB colonization but without VAP; CRAB-I, CRAB VAP patients; WBC, White blood cell; NEU, Neutrophils; Hb, Hemoglobin; Plt, blood platelet; ALT, Alanine aminotransferase; AST, Aspartate aminotransferase; CRP, C-reactive protein; APACHE II, Acute Physiology and Chronic Health Evaluation II.

^a^ Patient history.

^b^ During the 30 days preceding patients’ specimen collection onset.

^c^ On the patients’ specimen collection day.

^d^ After the patients’ specimen collection date.

^e^ Data analysis of CRAB-C patients and CRAB-I patients.
