# Supplementary material for: Comparative Respiratory Tract Microbiome Between Carbapenem-Resistant Acinetobacter baumannii Colonization and Ventilator Associated Pneumonia
Source: Front Microbiol. 2022 Mar 4;13:782210. doi: 10.3389/fmicb.2022.782210 (PMC8931608; doi:10.3389/fmicb.2022.782210)
Supplement: Supplementary Material 1 — Comparative respiratory tract microbiome between Carbapenem-resistant Acinetobacter baumannii colonization and ventilator associated pneumonia. [file Data_Sheet_1.ZIP › Frontiers Supplementary/Supplementary information legends.docx]

**Supplemental Figure**

**Supplemental Figure 1.** Antimicrobial drug sensitivity test of *Acinetobacter baumannii* isolates collected in this study.

Note: TZP, Piperacillin/ tazobactam; CSL, Cefoperazone/sulbactam; CAZ, Ceftazidime; CRO, Ceftriaxone; FEP, Cefepime; CIP, Ciprofloxacin; LVX, Levofloxacin; IPM, Imipenem; MEM, Meropenem; TMX/SXT, Trimethoprim/sulfamethoxazole; AMK, Amikacin; GEN, Gentamicin; POL, Polymyxin; TGC, Tigecycline

**Supplemental Figure 2.** Rarefaction analysis of bacterial 16S rRNA sequences.

**(A)** Rarefaction analysis of bacterial 16S rRNA gene sequences among samples was used to evaluate whether further sequencing would likely detect additional taxa, indicated by a plateau. The subject sample is indicated by the colour key at the top right corner. (**B)** Rarefaction analysis of bacterial 16S rRNA gene sequences among three group. The subject sample is indicated by the colour key at the top right corner. The y axis denotes the number of operational taxonomic units detected by Miseq sequencing at the corresponding sequencing depths shown along the x axis.

**Supplemental Figure 3.** Comparison of microbial composition between the metagenomics and 16S rRNA data.

**(A)** Overlap of identified genera between two data sets. The genera per group are visualized in as circle brownish yellowfor metagenomics data, and in green for 16S rRNA data. (**B)** The top 10 most abundant genera in the metagenomics and 16S rRNA analysis in CRAB-N group, respectively. (**C)** The top 10 most abundant genera in the metagenomics and 16S rRNA analysis in CRAB-C group, respectively. (**D)** The top 10 most abundant genera in the metagenomics and 16S rRNA analysis in CRAB-I group, respectively.

**Supplemental Figure 4.** Comparison of phyla and genus within the LRT microbiota of the three groups using Metagenomics analysis.

**(A)** Comparison of the average abundance of each bacterial phylum in each of the CRAB-N control groups and CRAB-C, CRAB-I patients, respectively. (**B)** Comparison of the average abundance of each bacterial phylum in each patient, respectively. (**C)** Comparison of the average abundance of each bacterial genus in each group, respectively. (**D)** Comparison of the average abundance of each bacterial genus in each patient, respectively. The subject group is indicated by the colour key at the top right corner.

**Supplemental Figure 5.** Microbial alpha and beta diversity in the 16S amplicon and Metagenomics analysis of three groups.

**(A)** Box plots depict microbiome diversity differences according to the Shannon index, and Gini Simpson index between both CRAB negative groups and the CRAB positive group through 16S amplicon analysis. (**B)** Box plots depict Alpha diversity differences among three groups through metagenomics analysis. The upper and lower ranges of the box represent the 75% and 25% quartiles, respectively. (**C)** The x-axis shows PCo 1 and the y-axis PCo 2 form Bray-Curtis distances for β-diversity through 16S amplicon analysis. (**D)** The x-axis shows PCo 1 and the y-axis PCo 2 form Bray-Curtis distances through metagenomics analysis. The changes per patient are visualized in as dashed lines dark red for CRAB-I, in blue for CRAB-C and in gray for CRAB-N. Logistic regression analysis showed that the change of PCo1 was significantly lower in patients who developed pneumonia (p=0.002 and 0.001, respectively). Significant differences are indicated by *P<0.05, **P <0.01, ***P <0.001.

**Supplemental Figure 6.** PhyloGenetic characterizations of 46 *Acinetobacter baumannii* isolates.

**(A)** Average nucleotide identity (ANI) of 46 isolates. (**B)** Partial snapshot of *Acinetobacter baumannii* population analyzed using eBURST distance and PHYLOViZ 2.0 software. Groups are annotated as CCs. Abbreviations: CC: Clonal complex.

**Supplemental Table**

**Supplemental Table 1.** 16S rRNA gene sequences and Metagenomics Analysis sequences statistics of ETA and Whole genome sequencing analysis statistics of CRAB sequenced in this study.

**Supplemental Table 2.** Demographics and clinical parameters of patients enrolled in the study.

**Supplemental Table 3.** Anosim analyzes differences in community structure between groups.
