# Supplementary figures and images for: Comparative Respiratory Tract Microbiome Between Carbapenem-Resistant Acinetobacter baumannii Colonization and Ventilator Associated Pneumonia
Source: Front Microbiol. 2022 Mar 4;13:782210. doi: 10.3389/fmicb.2022.782210 (PMC8931608; doi:10.3389/fmicb.2022.782210)

A

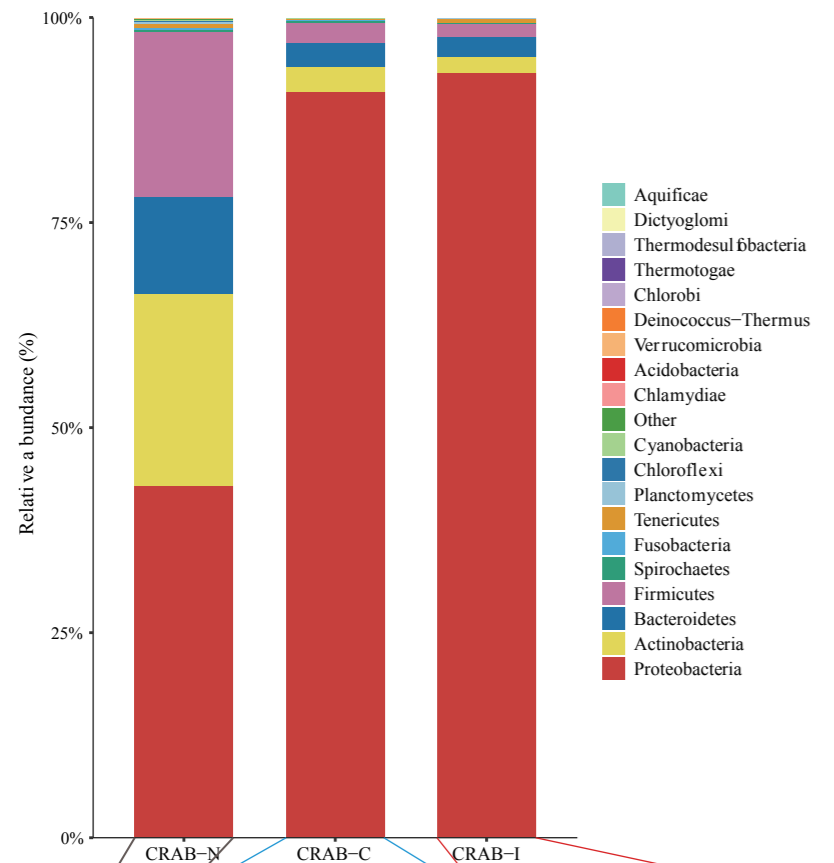

B

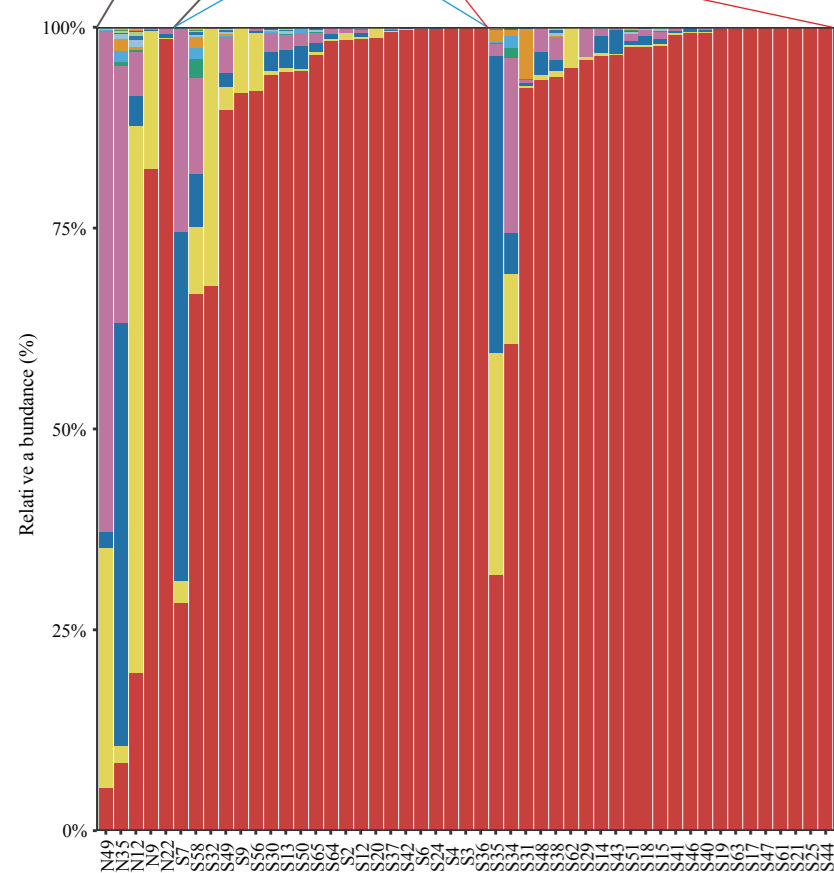

C

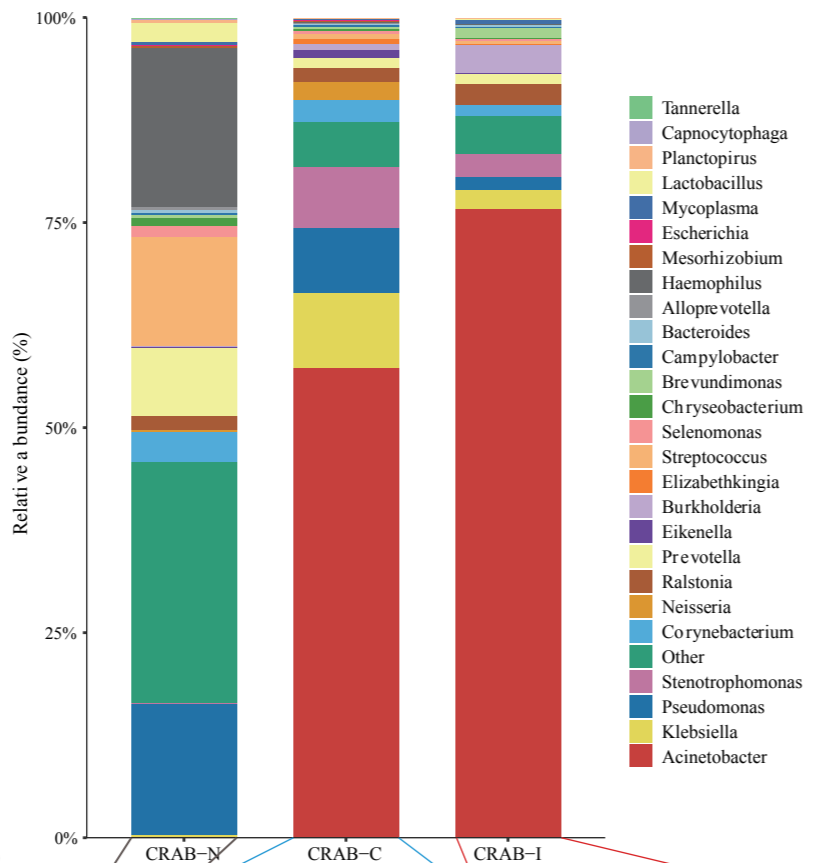

D

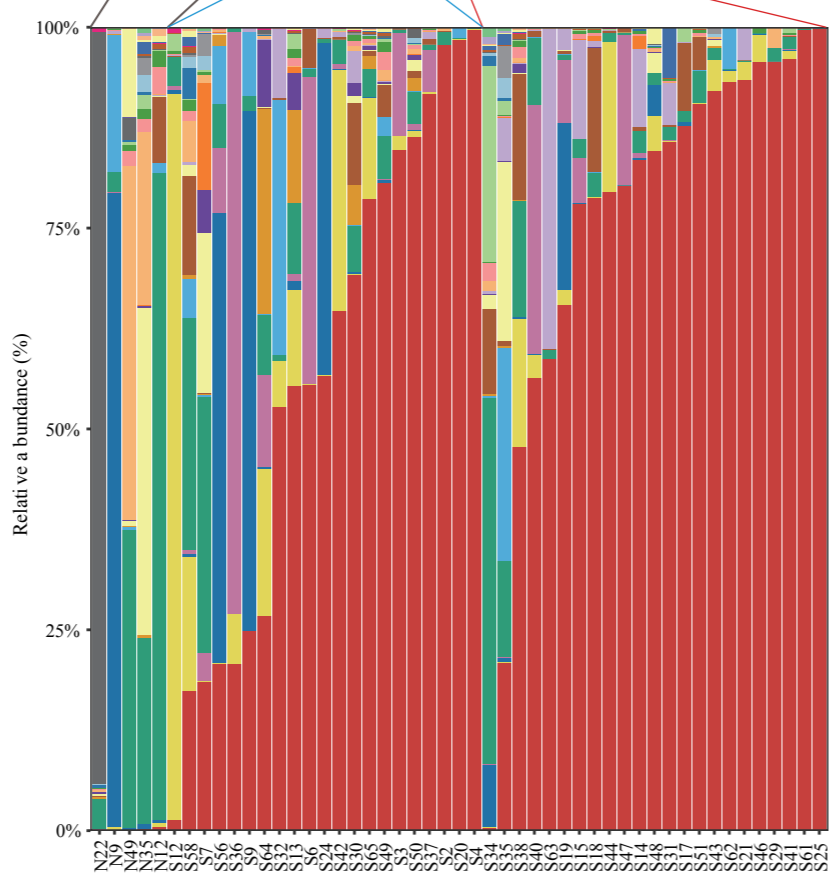

Supplement: Supplementary Material 1 — Comparative respiratory tract microbiome between Carbapenem-resistant Acinetobacter baumannii colonization and ventilator associated pneumonia. [file Data_Sheet_1.ZIP › Frontiers Supplementary/figs4.pdf]

**A1**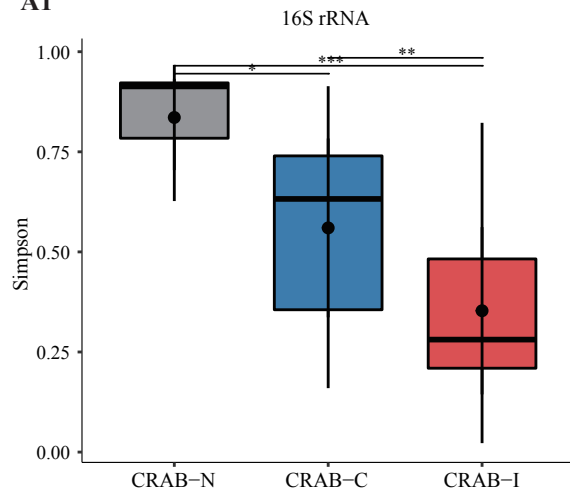**B1**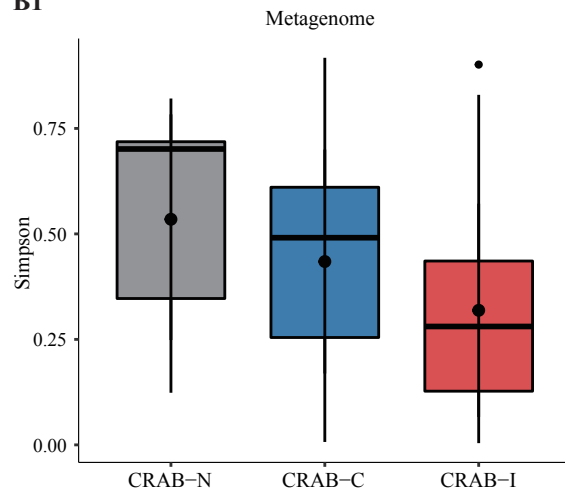**C**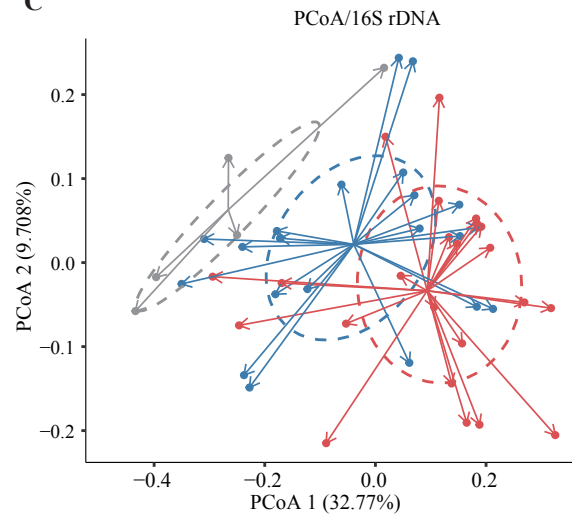**A2**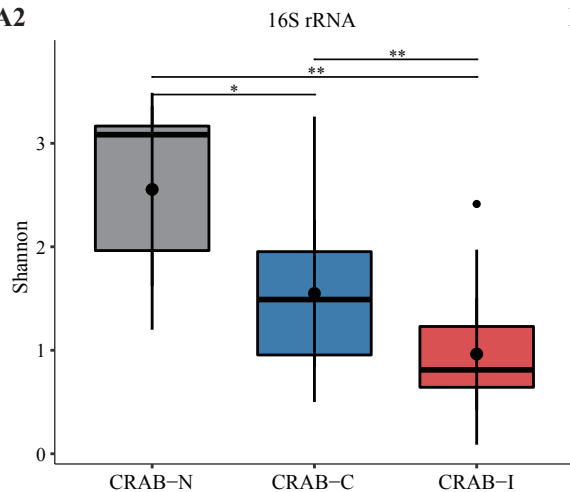**B2**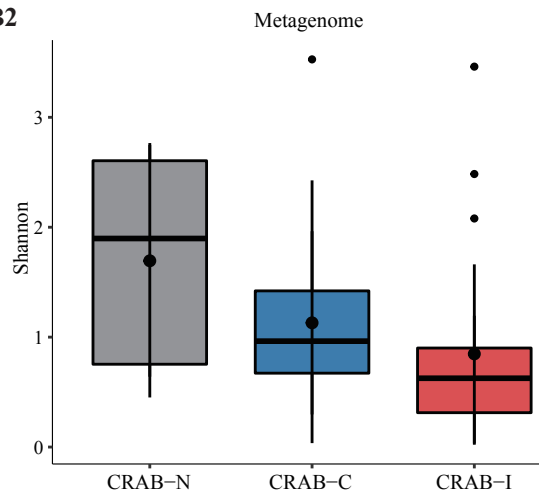**D**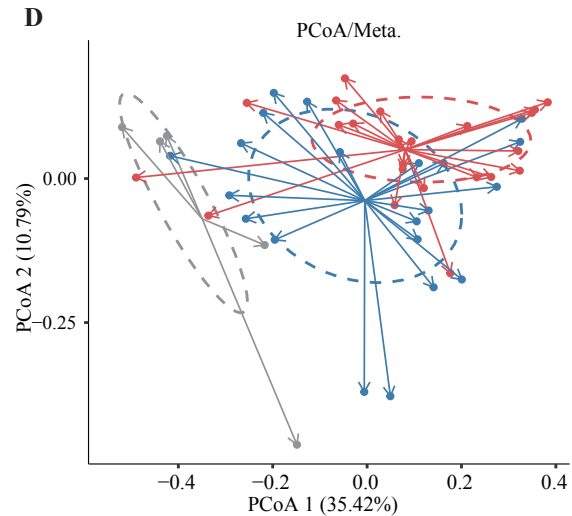

—●— CRAB-N    —●— CRAB-C    —●— CRAB-I

Supplement: Supplementary Material 1 — Comparative respiratory tract microbiome between Carbapenem-resistant Acinetobacter baumannii colonization and ventilator associated pneumonia. [file Data_Sheet_1.ZIP › Frontiers Supplementary/figs5.pdf]

A

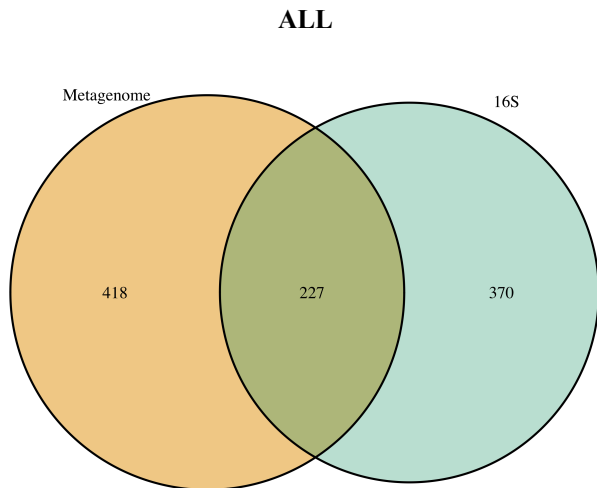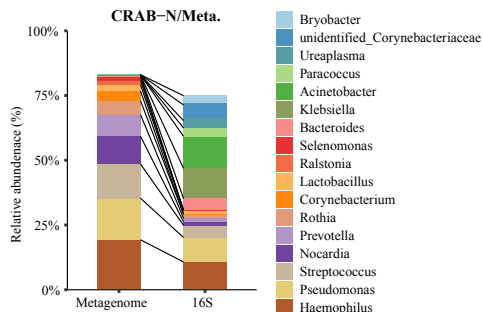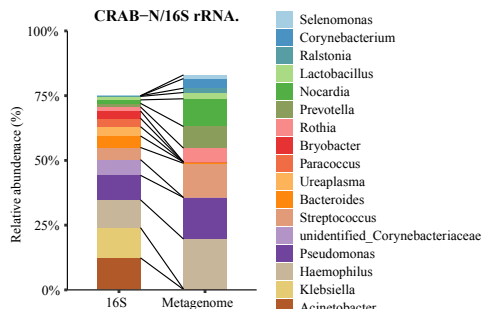

C

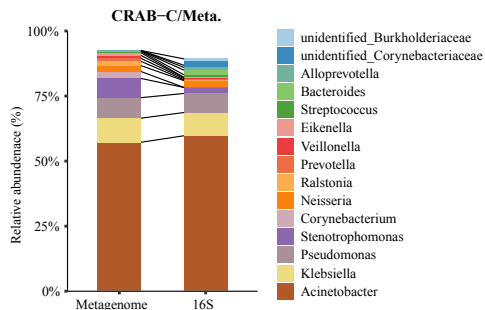

D

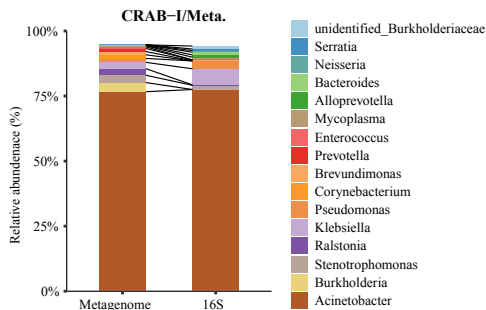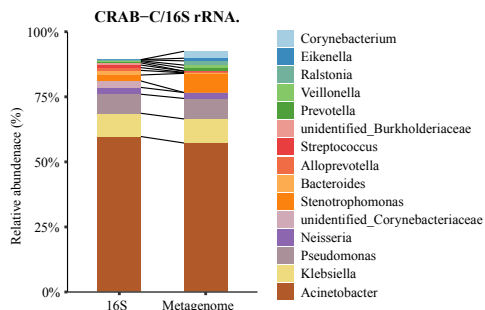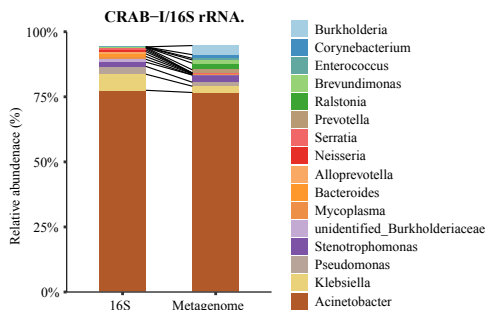

Supplement: Supplementary Material 1 — Comparative respiratory tract microbiome between Carbapenem-resistant Acinetobacter baumannii colonization and ventilator associated pneumonia. [file Data_Sheet_1.ZIP › Frontiers Supplementary/figs3(revised).pdf]

A

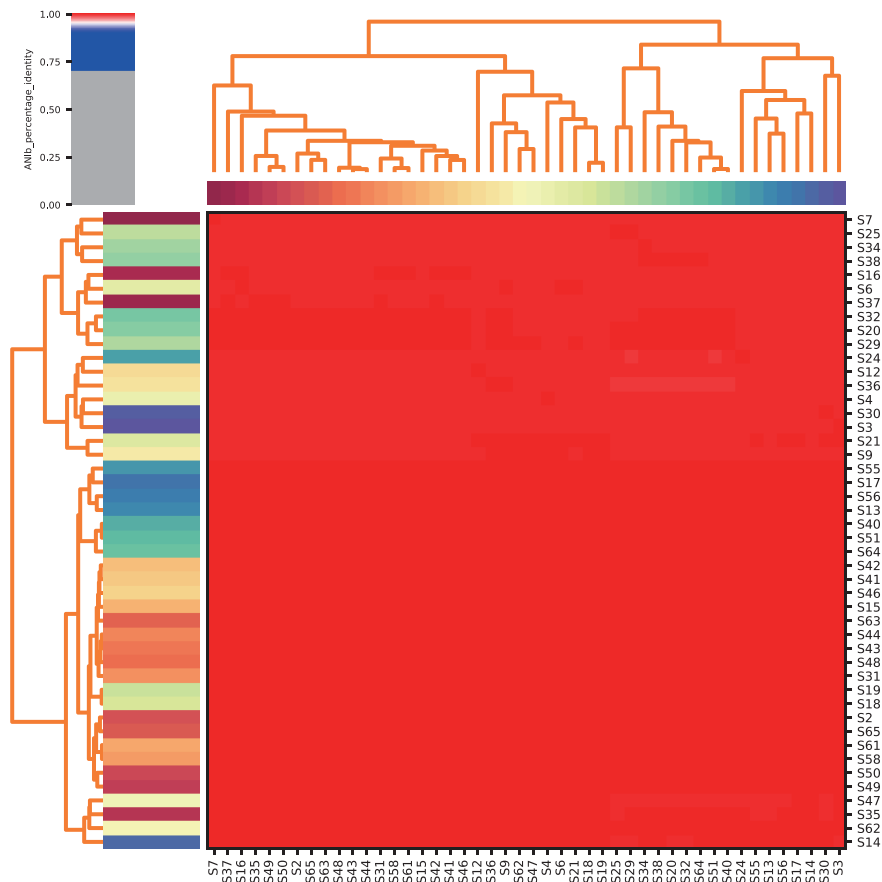

B

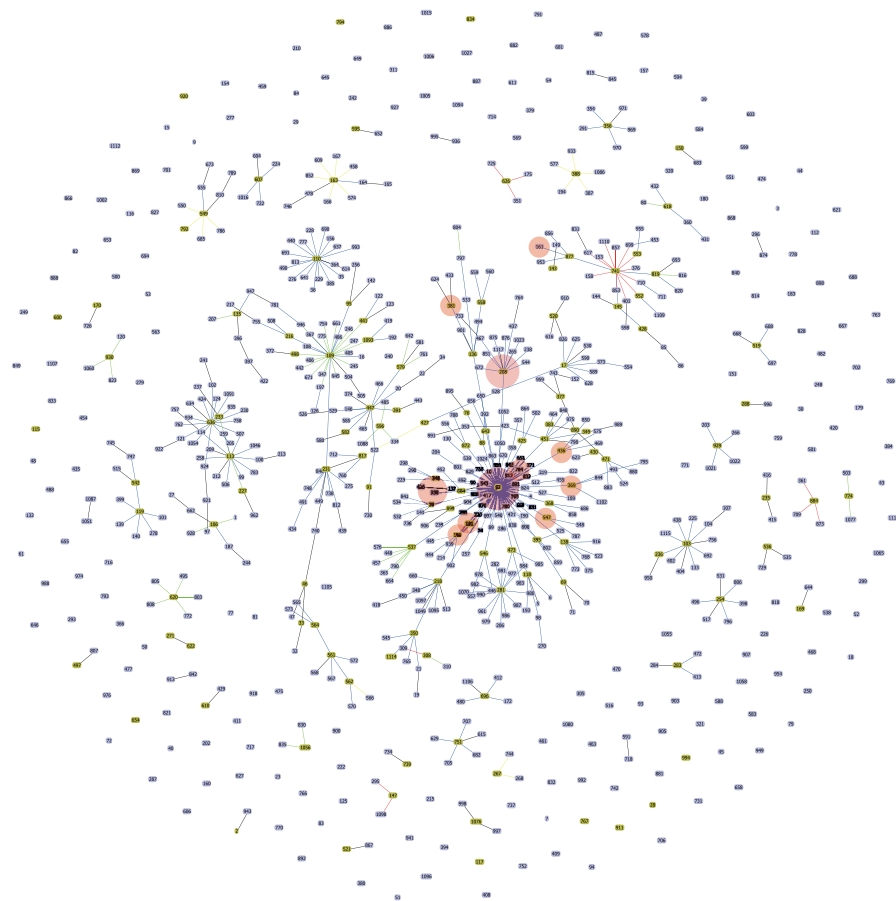

Supplement: Supplementary Material 1 — Comparative respiratory tract microbiome between Carbapenem-resistant Acinetobacter baumannii colonization and ventilator associated pneumonia. [file Data_Sheet_1.ZIP › Frontiers Supplementary/figs6.pdf]

**A**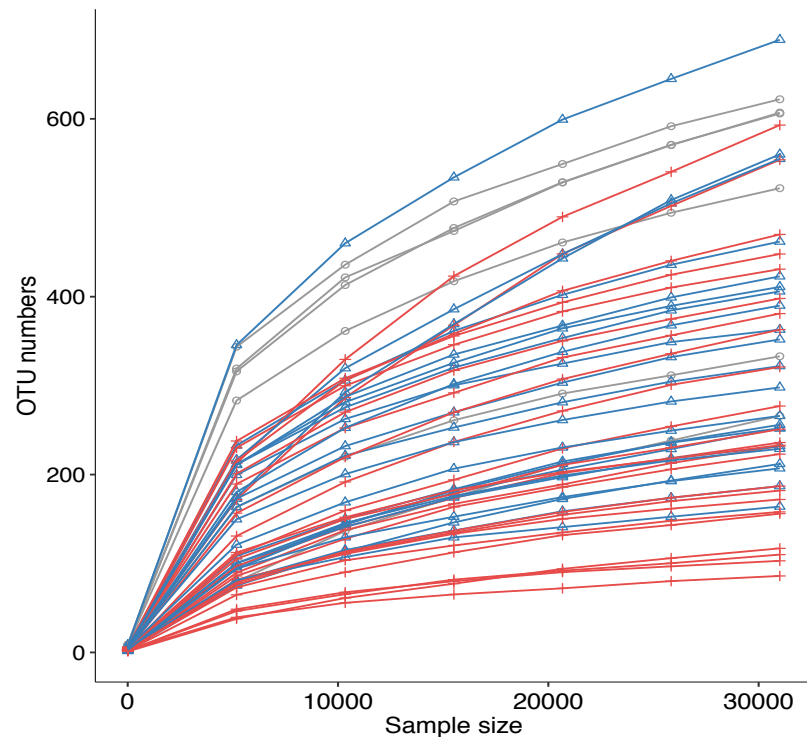**B**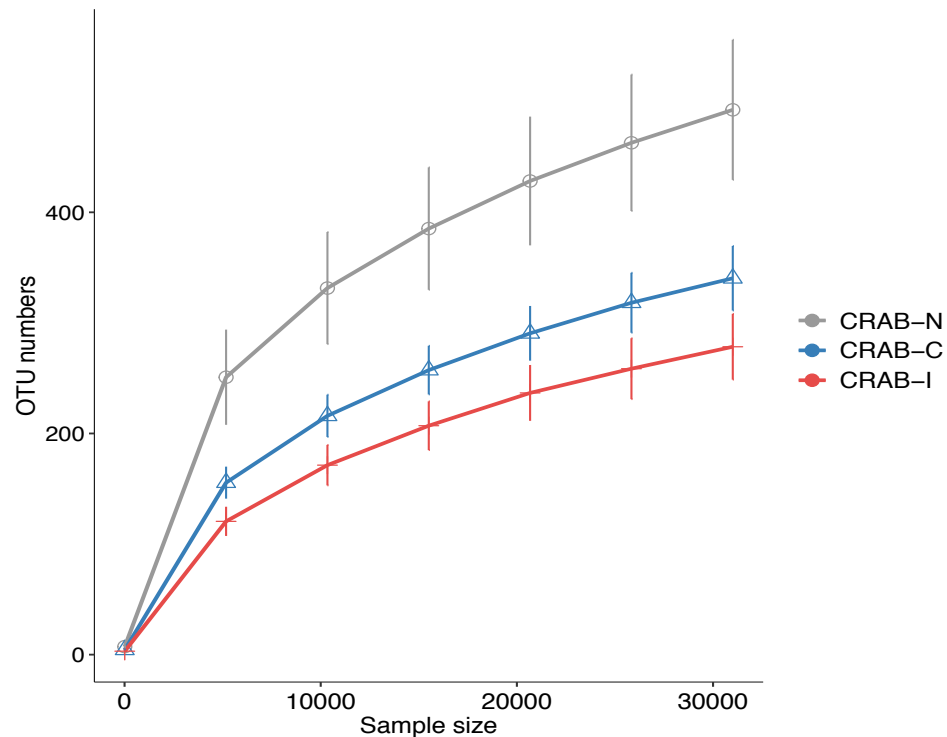

Supplement: Supplementary Material 1 — Comparative respiratory tract microbiome between Carbapenem-resistant Acinetobacter baumannii colonization and ventilator associated pneumonia. [file Data_Sheet_1.ZIP › Frontiers Supplementary/figs2.pdf]

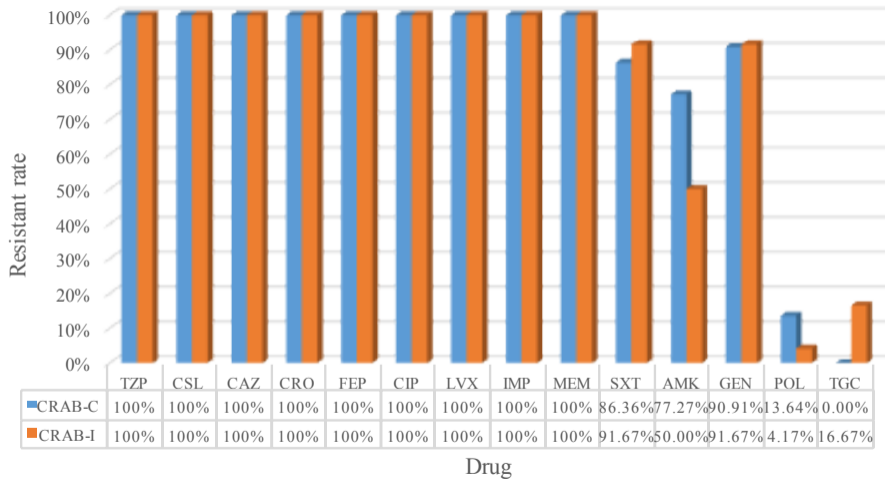

Supplement: Supplementary Material 1 — Comparative respiratory tract microbiome between Carbapenem-resistant Acinetobacter baumannii colonization and ventilator associated pneumonia. [file Data_Sheet_1.ZIP › Frontiers Supplementary/figs1.pdf]
